# Validation of a simplified oral indicator for home care nurses to refer older people to dental care professionals

**DOI:** 10.2340/aos.v83.42487

**Published:** 2024-12-16

**Authors:** Lina F. Weening-Verbree, Annemarie A. Schuller, Wim P. Krijnen, Cees P. van der Schans, Sytse U. Zuidema, Johannes S. M. Hobbelen

**Affiliations:** a Research Group Healthy Ageing, Allied Health Care and Nursing and FAITH Research, Hanze University of Applied Sciences, Groningen, The Netherlands; b Center for Dentistry and Oral Hygiene, University Medical Center Groningen, Groningen, The Netherlands; c TNO the Netherlands Organisation for Applied Scientific Research, Leiden, The Netherlands; d Faculty of Science and Engineering, University of Groningen, Groningen, The Netherlands; e Department Health Psychology, University Medical Centre Groningen, Groningen, The Netherlands; f Department Rehabilitation Medicine, University Medical Centre Groningen, Groningen, The Netherlands; g Department of Primary and Long-Term Care, University of Groningen, University Medical Center Groningen, Groningen, The Netherlands

**Keywords:** Access to dental care, older people, dental triage, oral health assessment, home care nursing

## Abstract

**Objectives:**

This study aims to explore the identification of older people in need of dental consultation, with a Simplified Oral Indicator (SOI) used by home care nurses (HCNs) and with the Geriatric Oral Health Assessment Index (GOHAI-NL) completed by older people themselves, compared with the Oral Health Assessment Tool (OHAT-NL), performed by dental hygienists.

**Methods:**

The HCNs completed SOI based on their professional view, knowledge and experience; scores red/orange/green were given to older people for oral health and oral hygiene. Older people completed the GOHAI-NL and dental hygienists completed the OHAT-NL.

**Results:**

Data from 141 older people were analysed. Sensitivity and specificity of SOI –OHAT-NL were low (0.45 and 0.64, respectively); SOI scored only few older people as ‘red’, while only 11 older people did not need a dental referral according to the OHAT-NL. OHAT-NL and GOHAI-NL correlation was significant, but low (*r* = -0.226, *p* = 0.012).

**Conclusion:**

Simplified Oral Indicator is currently not sensitive enough to identify older people in need of dental consultation. Additional education to HCNs and/or adjusting SOI may be needed. The GOHAI-NL seems not useful in dental triage.

## Introduction

As people age they can become care dependent or cognitively impaired, but with the support of formal home care nursing they can stay at their own homes in the community [1–3]. It is shown that older people who reside in their community have not visited a dentist for many years and their oral health is poor when they are admitted to a nursing home [4]. One explanation is that these older people do not seek professional dental help or dental care [5, 6]. Barriers to attend dental check-ups are reported as: a lack of transportation, not being insured for dental care or not being able to afford dental care, or being too frail or too ill to prioritise dental consultation [6–8]. Dental care provided by dental care professionals at older peoples’ home tends to be complex and is not often available [9–11]. Therefore, it is important that there is a focus on prevention and early detection of oral problems, by other health care professionals, for this particular group of older people [9–11].

Formal home care nurses (HCNs) are involved in activities of daily living (ADL) support of older people [12]. Formal HCNs are well placed to be increasingly involved in the prevention and monitoring of oral health. When necessary, HCNs should be able to refer older people to dental care professionals [12]. Professional advice of HCNs or the referral of an older client to a dental professional, can therefore be a key factor in getting older people to visit a dental professional [12, 13]. However, when it comes to referral to dental care professionals, it is known that it is a difficult matter for non-dental professionals [14–16]. For instance, non-dental care professionals tend to underestimate soft tissue problems and overestimate oral hygiene aspects or tooth wear, when completing oral assessment instruments in older people [15, 16]. Research indicates that registered nurses and other non-dental health care professionals need additional training and instruction to differentiate between pathology and age-related dental aspects, and to complete these instruments [15–18]. The time needed to complete an actual oral assessments and the additional education needed to complete intra oral assessments with sufficient quality are barriers for non-dental health care professionals [14–16, 19].

Instruments that are used often in the literature are the Revised Oral Assessment Guide (ROAG and the Oral Health Assessment Tool (OHAT) [16–20]. These instruments measure the oral health and hygiene of older people both with and without natural teeth, through an intra oral assessment and are valuable. The OHAT and ROAG were originally designed to be used by non-dental care professionals, but they are now also used by dental care professionals [21–24]. However, Dutch HCNs are not trained to thoroughly inspect the mouth of the older people they care for and the majority of older people performed their own daily oral care [25].

It is shown that older people have considerable preventive and curative dental care needs [6, 7, 26], and therefore dental referral is needed. Older people themselves are not aware of oral health problems, such as dry mouth, pain, and difficulty with chewing [6, 7, 26]. Oral health is rated higher by older people themselves than when viewed by oral health professionals [27, 28]. A self-assessment questionnaire for older people’s oral health was considered a valuable indicator for self-assessed oral health; the Geriatric Oral Health Assessment Index in Dutch language (GOHAI-NL) [28, 29]. It showed the discrepancy between perceived oral health by older people and observed oral health by dental professionals [28, 29]. This highlights the importance of notifying oral problems and advising older people to visit dental care professionals. To date GOHAI or GOHAI-NL were not used as an instrument to refer or to advice older people, to dental care professionals.

Given the above, it can be said that HCNs, involved in the daily care of community-dwelling frail older people, could refer older people to dental care professionals. These HCNs need a simple and not too time-consuming triage instrument which should not require additional training or education. As part of an implemented ‘Oral Care Program’ (OCP) to improve the daily oral care of frail, home-dwelling older people, a ‘Simplified Oral Indicator’ (SOI) was introduced [25]. The SOI aims to be a simple tool for HCNs to indicate older people’s oral health and/or oral hygiene, in order to refer older people to a dental care professional for consultation and/or treatment. The implemented OCP also included self-assessments of oral health of older people’s oral health with the GOHAI-NL and an oral health screening performed by dental hygienists, using OHAT in Dutch language.

The aims of the current study were to explore the diagnostic accuracy of a SOI performed by HCNs to refer older people to dental care professionals as well as to explore the relationship between assessment of oral health performed by dental hygienists and self-assessed oral health by older people, with GOHAI-NL.

## Materials and methods

### Design

A cross-sectional and explorative study.

### Sample and recruitment

This study was part of a larger study regarding the implementation of an OCP in a home care nursing setting [25]. Six home care organisations in the northern part of the Netherlands participated in the implementation of OCP, with all agreeing to support their district nursing teams in joining the OCP [25]. In the Netherlands, nursing staff with different levels of training work in home care. In this study, we have included all home care nursing team members, with various levels of training. In this study, HCNs is used as a collective term for nursing team members. Part of the OCP was using three different quantitative instruments to assess oral health in community-dwelling frail older people: a SOI to rate the older people’s oral health and hygiene, performed by HCNs, clinical oral health of older people assessed by dental hygienists, and self-assessed oral health by older people themselves.

The participating home care nursing teams were asked to approach the older people they care for; eligible participants. Inclusion criteria were: all people 70 years or older who were using formal home care. The level of formal home care is determined at an intake visit by a registered community nurse in a formal home care organisation [12]. Depending on the care demands, care dependency of the older person and sustained indication of care support, formal HCNs may support older people with ADL care or specific nursing care [30].

Exclusion criteria were being legally incapable of giving informed consent or being too ill to participate in the study. Participants were only included in the analyses if all three measurements were completed at baseline.

From all the older people approached by their HCNs, 190 clients agreed to participate in the study and provided informed consent. After informed consent was given, 18 participants could not be examined by the dental hygienist because it was too difficult to make an appointment with the participants or the participants did not want to be visited by someone they did not know, or they became too ill. Another 31 participants were excluded from the analysis, because only OHAT-NL data were available, or the older people joined the study after the baseline measurements were completed.

### Ethical considerations

All older people involved in this study, signed an informed consent form. All data were processed anonymously and privacy was respected, according to the requirements of the Personal Data Protection Act. No ethical approval was needed according to the Medical Ethical Committee of the University Medical Center Groningen for this study (study number 201700693). This study has been conducted in full accordance with the Declaration of Helsinki. Between January 2018 and September 2019, data were collected.

### Assessment instruments

At baseline, before implementing OCP, the three instruments of this study, were completed for the older people who were included: a SOI to rate the older people’s oral health and hygiene performed by HCNs, a clinical oral assessment of the older people performed by dental hygienists, and a self-assessment on oral health by older people themselves.

#### Simplified Oral Indicator

Prior to the first measurements of oral health (care) by the dental hygienists, the HCNs completed the SOI (Appendix 1). The HCNs were asked to evaluate the oral health and oral hygiene of the older people on a three-point scale:

- green score when oral health and hygiene is ‘adequate’ (code 0)
- orange score when oral health and hygiene is ‘doubtful’ (code 1)
- red score when oral health and hygiene is ‘inadequate’ (code 2)

No additional education was provided before HCNs completed SOI. The completion of SOI was based solely on the HCNs’ professional judgement, tacit knowledge, and personal experience with oral health/hygiene of older people. No additional instructions were given and the HCNs did not necessarily assess the older persons’ mouth. Home care nurses made use of what they knew about their clients, the clients’ oral care habits or what their professional view was on the clients’ oral health and hygiene. Since HCNs are involved in daily nursing care of older people, they may have a professional opinion about their clients’ oral health care.

An orange or red score was considered to ‘refer the client to a dental professional’, because the HCN is in doubt or oral health or hygiene is inadequate and a dental care professional should see the older person. A detailed description of HCNs who completed the SOI in older people was given in an earlier report [25].

#### Oral health assessment tool

Dental hygienists completed the OHAT, which was translated to Dutch, in OHAT-NL (Appendix 1). The OHAT is a valid and reliable instrument to assess the oral status of older people and can be used for clients with both natural teeth and dentures, and is also used in other studies by dental professionals to measure oral health and hygiene [20, 24, 31, 32]. Pearson correlations between OHAT categories: lips, tongue, gums and tissues, natural teeth, dentures, and associated dental examination findings on these categories varied between 0.60 and 1.0 [20]. Although OHAT was originally developed to be used by non-dental health care professionals, according to different studies, nursing home staff need training and education to complete the OHAT accurately [16, 19].

The OHAT consists of eight items, reflecting the total oral health and oral hygiene condition of an older person and was therefore used in this study as ‘reference measurement’, completed by dental hygienists. The condition of the lips, tongue, gums and tissues, saliva, natural teeth (if present), dentures (if present), oral cleanliness, and dental pain are scored on an ordinal score (0 = healthy, 1 = oral changes, or 2 = unhealthy). The items ‘natural teeth’ and ‘dentures’ are only scored, if applicable. The sum scores of OHAT may vary between 0 and 16. A lower score indicates a better oral health. In the original OHAT description, a client should be referred to a dental professional, when any item is scored with ‘1’or ‘2’; a sum score larger than zero [20]. The sum scores of OHAT can thus be dichotomised: sum scores >0 dental referral is needed and sum scores 0 no dental referral is needed ‘no referral needed’. Additionally, the dental hygienists completed a short questionnaire about the older people; including age, gender, dental visits (yes/no), dental status (in four categories: natural teeth/dentures/natural teeth and dentures/dentures on implants), and daily oral hygiene routines.

Seven registered dental hygienists with experience of working in the field of gerodontology were assigned to administer the OHAT (Dutch version) of each older participant at baseline. The dental hygienists practised and standardised the completion of OHAT-NL during a single meeting. The oral assessments were performed in the clients’ homes while they were sitting in a chair or bed. Ambient light was used to assess the oral cavity.

#### Geriatric Oral Health Assessment Index-NL

The GOHAI-NL is a questionnaire with 12 items for older people about their oral health, to complete themselves [29] (Appendix 1). After signing informed consent, but prior to the first measurements of the dental hygienists, GOHAI-NL was completed by the participants. The GOHAI-NL is a reliable and validated self-assessment instrument to measure the perceived oral health by older people; Cronbach’s’ alpha was 0.80–0.86 and test-retest correlation 0.88–0.93 [29]. The GOHAI-NL consists of three domains: physical functioning (items 1, 2, 4), pain and discomfort (items 3, 5, 8 and 12), and psychosocial functioning (items 6, 7, 9, 10 and 11). The questions are scored by older people themselves on a Likert scale; never, seldom, sometimes, often, very often or always. The GOHAI-NL score is the sum of the item scores (score 1–5 per answer; total score from 12 to 60; 12 items). Higher values represent a more positively perceived oral health.

### Analyses

Descriptive statistics (frequency distributions, sum scores means [standard deviations]) were used to summarise the result of the SOI, OHAT-NL and GOHAI-NL, and boxplots for SOI and OHAT-NL were added to graphically present these results. The SOI scores were dichotomised in all analysis: ‘Green’ was scored not to refer, and ‘Orange’ and ‘Red’ were scored as ‘to be referred to a dental professional’. The option ‘Orange’ was included, to give an option to HCNs in case they had doubt about the condition of the oral health or hygiene of the older person. Yet, ‘Orange’ was considered as ‘needs to be referred’ option, because it is not a ‘Green’ score. The OHAT-NL was used as reference standard to report oral health and hygiene [32].

Sensitivity and specificity values of the SOI were calculated using the OHAT-NL as reference standard.

Sensitivity (true positive rate) is the probability of a referral to a dental professional which is correctly identified by a HCN (SOI score red/orange), according to OHAT-NL by dental hygienists.

Specificity (true negative rate) is the probability of ‘do-not-refer to dental professional’ according to a HCN (SOI green score) being correct, when the OHAT-NL score is zero according to dental hygienists.

Furthermore, true prevalence values, positive predictive values (the percentage of SOI red/orange scores who were actually correctly referred according to OHAT-NL > zero), negative predictive values (the percentage of SOI green scores, that correctly not referred the older person according to OHAT-NL = zero), and 95% confidence intervals (CIs) were calculated.

Additionally, the sensitivity and specificity of the SOI with separate OHAT-NL items as references were calculated in order to investigate diagnostic accuracy in more detail. Again, positive predictive values, negative predictive values, and true prevalence values, with 95% CIs were calculated. Differences of the SOI indicated groups (red/orange versus green) with respect to the OHAT-NL were studied by an independent samples *t*-test. The frequency distributions on GOHAI-NL items were reported and sum scores were calculated. The GOHAI-NL item scores for questions 3, 5, and 7 were reverse-coded so that all items scored in the same direction. The cut-off values of GOHAI-NL are not described in the literature and therefore we explored the correlation between OHAT-NL and the GOHAI-NL sum scores, with Pearson’s correlations coefficient. A correlation coefficient between 0.7 and 1.0 was considered as strong and a correlation coefficient between 0.3 and 0.0 as negligible [33].

The level of significance was α = 0.05. All analysis were performed using Statistical Package for the Social Sciences for Windows Version 28.0 (IBM Corp., NY, USA) or using the statistical language R (version 4.1.0) and R studio 2022.12.0 [34].

## Results

### Sample characteristics

Characteristics of the 141 older people participating in this study are presented in Table 1. The mean age of the participants was 84 years (standard deviation [SD] 7.4 years); and 102 were women (72%). The SOI was completed by HCNs for 138 older participants and SOI group sizes are *n* = 76 in green (55%) and *n* = 62 (45%) in orange/red.

**Table 1:** Characteristics and oral status of older participants.

Characteristics	*n* = 141 (%)
Mean age (SD)	84 (7.4)
Gender, women	102 (72)
**Dental status**	
Natural teeth	19 (13)
Dentures	80 (54)
Dentures/natural teeth	32 (22)
Dental implants/dentures	16 (11)
Number of older people who do not visit a dental professional	79 (56)
**SOI**	***n* = 138 (%)**
Green	76 (55)
Orange	57 (41)
Red	5 (4)

SD: standard deviation; *n*: the number of participants the item is applicable to; SOI: Simplified Oral Indicator.

### Oral health assessment tool-NL and Simplified Oral Indicator

**Figure 1:**
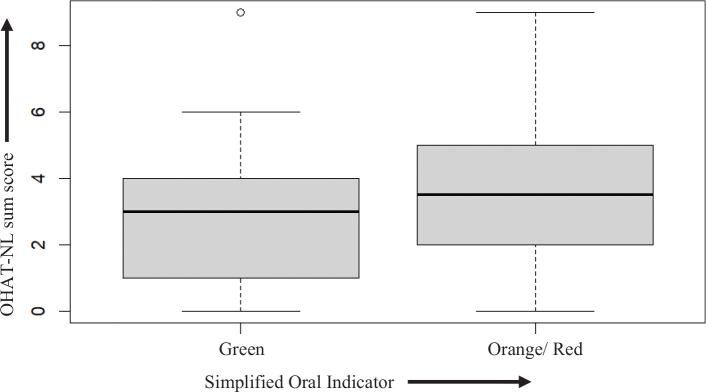
Boxplots of SOI and OHAT-NL sum scores. OHAT: Oral Health Assessment Tool; SOI: Simplified Oral Indicator.

The OHAT-NL sum scores could be calculated of *n* = 141 older people and the mean OHAT-NL score was 3.03 (SD 1.92) and ranged from 0 to 9. In Appendix 2, the distribution of the OHAT-NL scores is reported. It is shown that the lower OHAT-NL sum scores, which indicate a better oral health, are within the SOI ‘green’ category, presented in Figure 1. The boxplots show much overlap of OHAT-NL scores in green and orange-red groups. Also, it shows a large range of OHAT-NL scores in orange/red group, including low OHAT-NL scores, indicating a better oral health and hygiene.

The mean OHAT-NL sum score for the SOI ‘green’ group was 2.72 (SD 1.7) and the mean OHAT-NL sum score for the ‘red/orange’ group was 3.53 (SD 2.1). The independent samples *t*-test showed that this difference in means is statistically significant (*t* (116) *t* = -2.48; *p* = 0.017).

The dichotomised OHAT-NL sum scores show that some older people should not be referred based on OHAT-NL scores (Tables 2 and 3). The correctly ‘refer older person no’ scores are *n* = 7 (true negative cases) and the correctly ‘refer older person yes’ are *n* = 58 (true positive cases). The sensitivity of SOI–OHAT-NL total was 0.45 and specificity 0.64.

**Table 2:** OHAT-NL total, items and SOI cross tables.

**OHAT-NL total**	**OHAT – refer yes**	**OHAT – refer no**	**Total (*n*)**
SOI red – refer yes	58	4	62
SOI green – refer no	69	7	76
Total (*n*)	127	11	138
**OHAT-NL Lips**	**OHAT – refer yes**	**OHAT – refer no**	**Total (*n*)**
SOI red – refer yes	16	46	62
SOI green – refer no	25	51	76
Total (*n*)	41	97	138
**OHAT-NL Tongue**	**OHAT – refer yes**	**OHAT – refer no**	**Total (*n*)**
SOI red – refer yes	26	36	62
SOI green – refer no	31	45	76
Total (*n*)	57	81	138
**OHAT-NL Mucosa/gingiva**	**OHAT – refer yes**	**OHAT – refer no**	**Total (*n*)**
SOI red – refer yes	24	38	62
SOI green – refer no	17	59	76
Total (*n*)	41	97	138
**OHAT-NL Saliva**	**OHAT – refer yes**	**OHAT – refer no**	**Total (*n*)**
SOI red – refer yes	30	32	62
SOI green – refer no	39	37	76
Total (*n*)	69	69	138
**OHAT-NL Natural Teeth**	**OHAT – refer yes**	**OHAT – refer no**	**Total (*n*)**
SOI red – refer yes	16	8	24
SOI green – refer no	7	19	26
Total (*n*)	23	27	50
**OHAT-NL (partial) Prosthesis**	**OHAT – refer yes**	**OHAT – refer no**	**Total (*n*)**
SOI red – refer yes	23	31	54
SOI green – refer no	19	46	65
Total (*n*)	42	77	119
**OHAT-NL Oral hygiene**	**OHAT – refer yes**	**OHAT – refer no**	**Total (*n*)**
SOI red – refer yes	38	24	62
SOI green – refer no	41	35	76
Total (*n*)	79	59	138
**OHAT-NL Dental pain**	**OHAT – refer yes**	**OHAT – refer no**	**Total (*n*)**
SOI red – refer yes	13	49	62
SOI green – refer no	10	66	76
Total (*n*)	23	115	138

OHAT: Oral Health Assessment Tool; SOI: Simplified Oral Indicator.

**Table 3:** OHAT-NL total/items and SOI, sensitivity, specificity, true prevalence, positive and negative predictive values (95% confidence intervals).

OHAT-NL (item)	Sensitivity	Specificity	True prevalence	Positive predictive value	Negative predictive value
OHAT-NL total score	0.45 (0.36, 0.54)	0.64 (0.31, 0.89)	0.92 (0.86, 0.96)	0.94 (0.84, 0.98)	0.09 (0.04, 0.18)
OHAT-NL Lips	0.39 (0.24, 0.55)	0.53 (0.42, 0.63)	0.30 (0.22, 0.38)	0.26 (0.16, 0.38)	0.67 (0.55, 0.77)
OHAT-NL Tongue	0.46 (0.32, 0.59)	0.56 (0.44, 0.67)	0.41 (0.33, 0.50)	0.42 (0.30, 0.55)	0.59 (0.47, 0.70)
OHAT-NL Mucosa/gingiva	0.59 (0.42, 0.74)	0.61 (0.50, 0.71)	0.30 (0.22, 0.38)	0.39 (0.27, 0.52)	0.78 (0.67, 0.86)
OHAT-NL Saliva	0.43 (0.32, 0.56)	0.54 (0.41, 0.66)	0.50 (0.41, 0.59)	0.48 (0.35, 0.61)	0.49 (0.37, 0.60)
OHAT-NL Natural Teeth	0.70 (0.47, 0.87)	0.70 (0.50, 0.86)	0.46 (0.32, 0.61)	0.67 (0.45, 0.84)	0.73 (0.52, 0.88)
OHAT-NL (partial) Prosthesis	0.55 (0.39, 0.70)	0.60 (0.48, 0.71)	0.35 (0.27, 0.45)	0.43 (0.29, 0.57)	0.71 (0.58, 0.81)
OHAT-NL Oral hygiene	0.48 (0.37, 0.60)	0.59 (0.46, 0.72)	0.57 (0.49, 0.66)	0.61 (0.48, 0.73)	0.46 (0.35, 0.58)
OHAT-NL Dental pain	0.57 (0.34, 0.77)	0.57 (0.48, 0.67)	0.17 (0.11, 0.24)	0.21 (0.12, 0.33)	0.87 (0.77, 0.94)

OHAT: Oral Health Assessment Tool; SOI: Simplified Oral Indicator.

Cross tables and sensitivity, and specificity for SOI and the different OHAT-NL items are also presented in Tables 2 and 3. The true prevalence of OHAT-NL total score was 0.92, whereas for the individual items ‘lips’, ‘tongue’, ‘mucosa’, and ‘dental pain’, the true prevalence was between 0.17 and 0.41. The OHAT-NL item natural teeth showed the highest values on sensitivity and specificity: sensitivity was 0.70 and specificity 0.70.

The HCNs reported SOI green scores (false negative cases) in 69 older people, while these older people shown OHAT-NL scores higher than zero and should have been referred to a dental care professional. The mean OHAT-NL sum score of these false negative cases was 3.00 (SD 1.6) and range 1–9; with a frequency of scores 8 and 9 in only one older person. HCNs indicated in these cases that oral health/hygiene of older people was ‘good’, while in some of these older people the oral health/hygiene was rated as ‘poor’ or even ‘very poor’ by the dental hygienists.

### Oral health assessment tool-NL and Geriatric Oral Health Assessment Index -NL

Baseline GOHAI-NL measurements were completed by 122 older people and the scores on the different GOHAI-NL items are presented in Table 4. According to the GOHAI instructions, the items 3, 5, and 7 are reversed questions. The positive ends of the scale are marked with green colour and the negative ends of the scale are marked red. The sum scores of GOHAI-NL ranged from 27 to 60. The mean score was 50.3 (SD 7.7).

**Table 4:** GOHAI-NL frequency distributions on item level.

GOHAI-NL items (*n* = 122)	Never (%)	Seldom (%)	Sometimes (%)	Often (%)	Very often or always (%)
1. Limit the kinds of food	54	17	20	3	6
2. Trouble biting or chewing	30	19	24	12	15
3. Able to swallow comfortably	10	6	7	13	64
4. Unable to speak clearly	71	12	11	3	3
5. Able to eat without discomfort	12	13	15	18	42
6. Limit contact with people	78	16	4	1	1
7. Pleased with look of teeth	7	6	11	21	55
8. Used medication to relieve pain	70	15	12	3	0
9. Worried about teeth, gums or dentures	51	24	16	8	1
10. Self-conscious of teeth, gums or dentures	66	20	9	3	2
11. Uncomfortable eating in front of others	63	22	9	3	3
12. Sensitive to hot, cold or sweet foods	57	28	7	6	2

GOHAI: Geriatric Oral Health Assessment Index; Green colored: positive end of the scale; Red colored: negative end of the scale.

**Figure 2:**
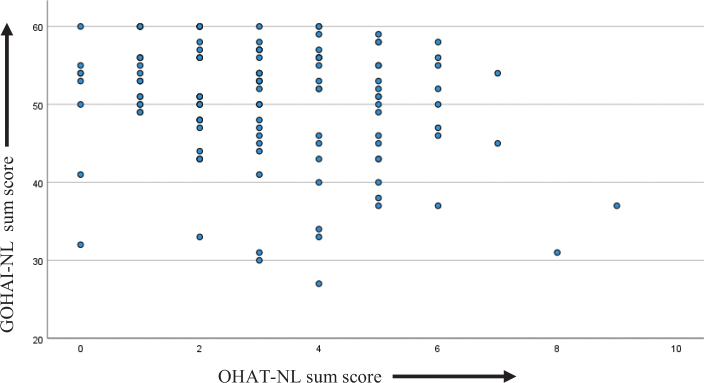
Scatterplot of OHAT-NL sum scores and GOHAI-NL sum scores. OHAT: Oral Health Assessment Tool; GOHAI: Geriatric Oral Health Assessment Index.

In Figure 2 a scatterplot of GOHAI-NL and OHAT-NL sum scores is shown and it visualises a weak negative correlation; a higher OHAT-NL sum score (more compromised oral health) correlates weakly with a lower GOHAI-NL sum score (a less well perceived oral health). The results of the Pearson correlation analysis revealed that this correlation was weakly negative, but statistically significant between older people’s clinical oral health (OHAT-NL by dental hygienists) and their self-assessed oral health (with GOHAI-NL), *r*(121), *r* = -0.226, *p* = 0.012.

## Discussion

In the current study, we compared measurements of dental hygienists with measurements of HCNs, while in other studies (also in the recent Dutch study on the OHAT [35]), often no dental professional measurements were used as ‘reference rater’ and only test-retest measurements are taken or intra-class-correlations of mutual nurses were calculated. The accuracy of SOI completed by HCNs, to refer older people to dental care professionals was explored. The SOI has a rather low sensitivity (0.45) and specificity (0.64). However, the SOI is possibly more accurate in its performance in older people with only natural teeth as it showed higher sensitivity and specificity values (both 0.70) on this particular item. Although the mean OHAT-NL scores between the SOI groups differ (*p* = 0.017), overall the findings have shown that SOI is currently not sufficiently accurate to support HCNs in referring older people to dental care professionals. Another aim of our study was to explore the relationship between assessment of oral health by dental hygienists and self-assessed oral health by older people. The OHAT-NL and GOHAI-NL scores showed low correlation and the sum scores of GOHAI-NL showed a wide range in perceived oral health by older people.

Remarkably, sensitivity and specificity values for natural teeth were higher in our study, which is in contradiction to the results of a recent study among Dutch community nurses on the Dutch OHAT, where it was shown that ‘natural teeth’ was a more difficult item for the HCNs to rate [35]. A possible explanation for this difference could be that in the current study, for the completion of SOI the HCNs did not necessarily have to inspect the oral cavity, while in the study to validate the OHAT for Dutch nurses [35] the HCNs did clinically inspect the oral cavity of older people, which is known to be difficult for non-dental care professionals [16]. Another explanation for higher sensitivity and specificity values on ‘natural teeth’ could be that oral health problems are more prompt in older people with natural teeth and therefore these problems are more observed by home care workers. This explanation corresponds to what we know about the oral health status of older people with natural teeth in this study sample: older people with natural teeth had worse oral health than older people with dentures. The study sample of the Dutch OHAT included only nine older persons with natural teeth [35] and thus comparison of study results may be biased by the small sample size.

Some items showed a low prevalence (‘lips’, ‘tongue’, ‘mucosa’, and ‘dental pain’) and were often scored with ‘zero’, meaning that no changes in lips, tongue, mucosa, and dental pain were seen; and this may have contributed to the low sensitivity and specificity values on these items. These low prevalences were also seen in another study in home dwelling older people [35].

The SOI – OHAT-NL sensitivity and specificity are both low and the HCNs have given a SOI score ‘red’ in only five older people and the majority of ‘false negatively not referred’ older people, were in the ‘orange’ group. Clearly, the number of older people with ‘red’ score was too low, one reason could be the possibility of scoring ‘orange’, which could have been an easy choice for HCNs, if they feel uncertain about a red score on the SOI. The low sensitivity value of 0.45 indicates that many older people with dental problems are missed by HCNs in dental triage. The specificity value of 0.64 reports on older people without dental problems who are correctly identified not to be referred. The implications of the low sensitivity are larger than the implications of the low specificity value; because in dental triage it is better to accidently refer too many older people than to miss older people that are in need of a dental professional. Our findings are in line with previous research showing that non-dental care professionals are more likely to underestimate dental problems in older people [15, 16]. Another possible explanation for the low sensitivity is that the OHAT-NL instrument is possibly oversensitive in its referral; it can be argued that the dental hygienists (with the completion of OHA-NL T) were too restrictive and may have overestimated the number of older people that should be referred to a dental care professional, since only 11 older people were given an OHAT-NL score of zero. Or, another possibility is that the OHAT is an instrument that is too rigid in itself and a sum score larger than zero is easily obtained, even with only dry lips for example. This could be a limitation of OHAT-NL and also, OHAT was originally designed to be used by non-dental care professionals [20]. The construct validity of OHAT is studied. It’s content was based on study of the literature but concurrent measures by dental professionals showed low and non-significant correlations on the items saliva (*r*(19) = 0.07) , oral cleanliness (*r*(19) = 0.15) and dental pain (*r*(19) = -0.1) [19, 20]. Even so, it should be mentioned that OHAT in itself is not flawless, regardless of the person completing OHAT.

The OHAT-NL and GOHAI-NL scores showed low correlation. The sum scores of GOHAI-NL showed a wide range in perceived oral health by older people; some older people scored particularly low, indicating that their perception of oral health was quite negative. One explanation for low correlation of GOHAI-NL and OHAT-NL could be that older people in general are more positive about their own dental health than dental professionals [27], and other explanation could be that clinically assessed oral problems do not automatically or immediately affect the perceived oral health [36], but this does not mean that dental treatment is not needed. If the older people in our study mention oral health problems in self-assessments, it is likely that oral health problems are present in reality and this emphasises the relevance of triage by HCNs in a frail population even more.

The mean GOHAI-NL sum scores in the current study are a bit lower than in another cohort of older people in the Netherlands [29]. This may because in the current study population, the proportion of edentulous people was higher and participants reported lower scores on the items about physical functioning and this is in line with other findings [37].

### Limitations

One of the limitations of our study is that SOI was not administered by more than one HCN for the same older person; we could not calculate intra-rater reliability. If more than one HCN would have completed the SOI for the same older person, an additional agreement analysis could have been performed (Cohen’s kappa for instance).

Another limitation is that with the completion of SOI, we did not ask to report the training level of the HCN that performed the SOI. It would have provided valuable insights on SOI’s performance in different training levels, and this could direct future education for nurses’ vocational training. On the other hand, all nursing staff in formal home care nursing teams, do have the same responsibility for oral care of older people, regardless what their educational background is. It can be argued that more practical educated nurses (often a shorter training level) are possibly more involved and engaged in individual clients’ daily care [38], and they may have more practical experience. Yet, 80% of the HCNs in our study received oral care education in their vocational training. However, 64% of the HCNs stated that they required more information on oral health [25] and possibly the HCNs in our study were well aware of ‘missing knowledge or skills’.

The relevance of these study results for other populations is possibly limited because in the current study, a rather small group of older people was studied and older people with dentures are over-represented in the study sample; which may have caused bias in terms that HCNs may find it easier to assess the oral health or hygiene in older people with dentures [39].

Because of the small study sample and the improvements that will be needed for the implementation and further study of SOI, this study could be seen as a pilot study of SOI as a triage instrument.

In this study we used OHAT-NL as the reference instrument for oral health and hygiene, because of its easy use for both dentate and edentulous older people; but OHAT-NL seems oversensitive in generating false positives considering referral to dental care professionals: if an older person has dry lips or a blister, it is scored as ‘1’ and immediate dental referral is needed according to the instructions of OHAT. We think that a consideration of different items should be taken into account, in making the referral. The OHAT-NL item ‘natural teeth’ showed higher sensitivity and specificity values, but obviously, this item cannot be completed for older people with dentures.

In this study we have considered orange and red scores on the SOI, completed by HCNs, reason to refer older people to dental professionals. It may be questioned whether referral is immediately necessary, when scoring orange or red. On the other hand, Dutch HCNs are not trained well enough to provide preventive dental care. In the Dutch dental care system, preventive care is provided by dental hygienists who work in private practices, supporting older people in daily oral care.

### Recommendations

Further research on the performance of the SOI by HCNs is needed and of dental triage by HCNs in general, it preconditions to accomplish correct triages, and also evaluate its use in daily practice. More attention should be paid to the HCNs who only referred few older people. It is preferable that the sensitivity is higher (older people referred by using SOI), because an incorrect dental referral (false positive) is not expected to be of major negative consequences for an older person in contrast to the expected negative consequences of a false negative score. One of the potential improvements of the implementation of SOI could be education about signs of dental problems or dental care neglect in older people. Possibly the SOI can be adjusted to two categories (green/red) to ‘force’ the HCNs to choose. No additional education and instruction were given to HCNs before the SOI was used in practice. Based on other studies [40, 41], it is expected that HCNs will be able to complete the SOI more accurately, if education about signals of oral problems in older people and when it is needed to refer to a dental care professional, will be provided. It is likely that HCNs do need education about oral care, additional to their vocational training, to use a simple instrument as SOI. Also, we have no clear understanding of the influence of the individual training level, knowledge or experience of the HCN who completed SOI. A follow-up study should take this into account.

Another future possibility is to gain insights in ‘what home care nurses’ look at?’ or how they ‘judge’ oral health or oral care of older people, if they do not actually look into older people’s mouth and to study what nurses need to assess the oral cavity?

Simplified Oral Indicator’s use and follow up of the HCN’s advice of the older people, should be studied, because in our study the older people were part of the implementation of an OCP that included an intraoral assessment by a dental hygienist, while it may be difficult for older people to find a dentist in the community, who accepts new patients [42, 43]. It is likely that not all older people need dental treatment from a dentist, but they would benefit from advice for daily oral care and hygiene from a dental hygienist who could also monitor their oral health. Therefore, visits to a dental practice for preventive dental care, should be encouraged.

Future implementation or testing SOI should therefore also include community dental practices, where older people are referred to. In another study, it was noticed that the majority of older people who were referred to dental care professionals by HCNs did not agree with the referral advice (e.g., older people did not find oral care important enough) and that no appointments were made, leading to undesirable delays [13]. Future implementation of SOI should include attention to raising awareness in older people, to motivate older people to adhere to the HCNs’ advice, and seek dental help on time.

## Conclusion

The SOI was studied in home care nursing setting, to serve as a triage instrument to refer older people with signs of dental problems. Currently, the SOI was not sensitive and specific enough to identify older people in need of dental referral; although, older people with natural teeth were more often correctly referred to dental professionals. Therefore, it is currently not advised to actually implement the SOI on a large scale without education for HCNs and/or adjusting SOI. Older peoples’ self-assessment seems not a useful addition in triage to dental care professionals.

## Supplementary Material

Validation of a simplified oral indicator for home care nurses to refer older people to dental care professionals

Validation of a simplified oral indicator for home care nurses to refer older people to dental care professionals

## Figures and Tables

**Table:** 

1.	Have made substantial contributions to conception and design, or acquisition of data, or analysis and interpretation of data	LWV, AS, WK, SZ, CS, HH
2.	Been involved in drafting the manuscript or revising it critically for important intellectual content;	LWV, AS, WK, SZ, CS, HH
3.	Given final approval of the version to be published. Each author should have participated sufficiently in the work to take public responsibility for appropriate portions of the content	LWV, AS, WK, SZ, CS, HH
4.	Agreed to be accountable for all aspects of the work in ensuring that questions related to the accuracy or integrity of any part of the work are appropriately investigated and resolved.	LWV, AS, WK, SZ, CS, HH

## Clinical trial registration number and name of trial register

No ethical approval was needed for this study, according to the Medical Ethical Committee of the University Medical Center Groningen (study name ‘Sûne Mûle’, study number 201700693).
