# Supplementary material for: Validation of a simplified oral indicator for home care nurses to refer older people to dental care professionals
Source: Acta Odontol Scand. 2024 Dec 16;83:42487. doi: 10.2340/aos.v83.42487 (PMC11707687; doi:10.2340/aos.v83.42487)
Supplement: Validation of a simplified oral indicator for home care nurses to refer older people to dental care professionals [file AOS-83-42487-s1.pdf]

Supplementary material has been published as submitted. It has not been copyedited or typeset by Acta Odontologica Scandinavica.

Appendix A -

SOI English and Dutch

OHAT-NL and Questionnaire English and Dutch

GOHAI-NL

Simplified Oral Indicator

Please complete on the dotted lines and mark what is applicable to the client

Name of client ……………………………………………………………………………………

Date of birth client ……………………………………………………………………………

Assessment of oral health / oral care Red / Orange / Green

Traffic light

RED

Oral care / Oral health inadequate

ORANGE

Oral care / Oral health doubtful

GREEN

Oral care / Oral health adequate

Simplified Oral Indicator

Graag antwoord invullen op de stippellijnen of omcirkelen wat van toepassing is

Naam cliënt ……………………………………………………………………………………………

Geboortedatum cliënt ……………………………………………………………………………

Beoordeling mondgezondheid / mondzorg Rood / Oranje / Groen

Stoplicht

ROOD

Mondzorg / mondgezondheid niet goed

ORANJE

Mondzorg / mondgezondheid twijfelachtig

GROEN

Mondzorg / mondgezondheid goed

Sûne Mûle

OHAT - NL

Categorie	0= gezond	1= verandering	2= ongezond	Score
**Lippen**	Glad<br>Roze<br>Vochtig	Droog<br>Schraal<br>Gebarsten of rode mondhoeken	Zwelling of bult op lip<br>Wit/rode/zwerende plek<br>Bloedende en/of zwerende mondhoeken	
**Tong**	Normaal<br>Vochtig<br>Roze<br>Ruw	Vlekkerig<br>Kloofjes aanwezig<br>Rood<br>Beslagen	Rode en/of witte plek<br>Zwerend en/ of gezwollen tong	
**Tandvlees + slijmvliezen**	Roze<br>Vochtig<br>Geen bloeding<br>Glad	Droog<br>Glanzend, ruw, rood, Gezwollen<br>Zweer onder prothetische voorziening	Gezwollen, gemakkelijk bloedend<br>Zweer<br>Wit en/ of rode plekken, algehele roodheid slijmvlies onder prothetische voorziening	
**Speeksel**	Vochtige slijmvliezen,<br>Waterig en vrij vloeiend speeksel	Droge, kleverige slijmvliezen<br>Kleine hoeveelheid speeksel<br>Cliënt ervaart een droge mond	Slijmvliezen uitgedroogd en rood<br>Weinig tot geen speeksel<br>Dik speeksel<br>Cliënt ervaart een droge mond	
**Natuurlijke tanden en kiezen (ja/nee)**	Geen cariës<br>Geen afgebroken tanden of kiezen<br>Geen wortelresten	1 - 3 carieuze of afgebroken tanden/ kiezen/ wortelresten<br>Slijtage van de tanden/ kiezen	Meer dan 4 carieuze of afgebroken tanden/ kiezen/ wortelresten<br>Ernstige slijtage van het gebit<br>Minder dan 4 tanden/ kiezen aanwezig in de mond	
**Prothetische voorziening (ja/nee)**	De prothetische voorziening is helemaal heel<br>Wordt regelmatig gedragen	1 deel van de prothetische voorziening of een tand/kies gebroken<br>Prothetische voorziening wordt slechts 1-2 uur per dag gedragen<br>Loszittende prothetische voorziening	2 of meer delen van de prothetische voorziening of tanden/kiezen van de voorziening zijn gebroken<br>Prothetische voorziening ontbreekt<br>Prothetische voorziening wordt niet gedragen<br>Prothetische voorziening zit los en plakmiddelen zijn nodig	
**Mondhygiëne**	Schoon<br>Geen etensresten, plaque of tandsteen in de mond of onder/op prothetische voorziening	Etensresten, tandsteen, plaque in 1-2 gebieden van de mond of op/ onder een klein deel van de prothetische voorziening<br>Slechte adem	Etensresten, tandsteen, plaque in groot deel van de mond of op de prothetische voorziening<br>Erg slechte adem	
**Dentale pijn**	Geen gedragsmatige, verbale of fysieke signalen voor pijn in de mond	Signalen van pijn in mond:<br>- verbaal<br>- gedragsmatig, zoals aan het gezicht zitten, op de lippen bijten, niet eten, agressie	Signalen van pijn in mond:<br>- verbaal<br>- gedragsmatig, zoals aan het gezicht zitten, op de lippen bijten, niet eten, agressie<br>Én ook fysieke aanwijzingen van pijn (zwelling van wang of tandvlees, gebroken tanden of zweren)	
○ Doorverwijzen naar een tandarts of mondhygiënist bij invullen van score 1 of 2 bij 1 van de items<br>○ Cliënt of verzorger/familie weigert tandheelkundige behandeling of doorverwijzing<br>○ Vul een MondzorgPlan in en start mondhygiëne behandeling<br>○ Retour voor evaluatie van de mondgezondheid op ..-..-....				**Totaal score:**<br><br>**/16**

Questionnaire Oral Care Older People

What kind of teeth does the client have? *Multiple answers possible*

◯ natural teeth upper jaw ◯ natural teeth lower jaw
◯ full prosthesis upper jaw ◯ full prosthesis lower jaw

Does the client visit a dentist? ◯ Yes ◯ No

If yes, when was the last visit to the dentist?

Number of months ago ◯ 0-6 ◯ 6-12 ◯ 12-24 ◯ different, namely …………………….

Does the client visit a dental hygienist? ◯ Yes ◯ No

If yes, when was the last visit to the dental hygienist?

Number of months ago ◯ 0-6 ◯ 6-12 ◯ 12-24 ◯ different, namely …………………….

Who performs daily oral care of the client? *Multiple answers possible*
◯ client ◯ informal caretaker ◯ home care nurse ◯ different, namely …………………….

What (daily) oral care is performed and how often is this done ?

Brushing teeth	◯ 2x day	◯ 1x day	◯ every other day	◯ different,………………
Interdental brushes	◯ 2x day	◯ 1x day	◯ every other day	◯ different,………………
Tooth picks (wood)	◯ 2x day	◯ 1x day	◯ every other day	◯ different,………………
Cleansing prosthesis	◯ 2x day	◯ 1x day	◯ different,………………	

How is the prothesis cleansed?

◯ toothpaste ◯ cleaning agent ◯ vinegar ◯ different,……………………………………………

How is the prothesis kept (during the night)?

◯ in water ◯ in prosthesis container ◯ different,…………………………………………….

Sûne Mûle

Questionnaire Oral Care Older People (Dutch)

Wat voor gebit heeft de cliënt? *Meerdere antwoorden mogelijk*

○ natuurlijk gebit boven ○ natuurlijk gebit onder ○ prothese boven ○ prothese onder

Bezoekt de cliënt een tandarts? ○ Ja ○ Nee

Indien ja, wanneer was het laatste bezoek aan de tandarts?

aantal maanden geleden ○ 0-6 ○ 6-12 ○ 12-24 ○ anders nl ……………………..

Bezoekt de cliënt een mondhygiënist? ○ Ja ○ Nee

Indien ja, wanneer was het laatste bezoek aan de mondhygiënist?

aantal maanden geleden ○ 0-6 ○ 6-12 ○ 12-24 ○ anders nl ……………………..

Wie voert de mondzorg uit? *Meerdere antwoorden mogelijk*

○ cliënt ○ mantelzorger ○ thuiszorgmedewerker ○ anders nl ……………………..

Welke mondzorgactiviteiten worden uitgevoerd en hoe vaak ?

Tandenpoetsen ○ 2x daags ○ 1x daags ○ om de dag ○ anders nl ……………………..

Ragen ○ 2x daags ○ 1x daags ○ om de dag ○ anders nl ………………………..

Tandenstoken ○ 2x daags ○ 1x daags ○ om de dag ○ anders nl ………………………..

Prothese schoonmaken ○ 2x daags ○ 1x daags ○ anders nl ……………………………….

Waarmee wordt de prothese schoongemaakt?

○ tandpasta ○ schoonmaakmiddel ○ azijn ○ anders nl ………………………………………

Hoe wordt de prothese weggezet?

○ in water ○ in prothesebakje ○ anders nl ………………………………………………………

GOHAI-NL

1. Hoe vaak hebt u de keuze van wat u eet, of de hoeveelheid die u eet, beperkt vanwege problemen met uw tanden of kunstgebit?

□ nooit □ zelden □ af en toe □ vaak □ (bijna) altijd

2. Hoe vaak hebt u last gehad bij het bijten of kauwen van eten, zoals taai vlees of appels?

□ nooit □ zelden □ af en toe □ vaak □ (bijna) altijd

3. Hoe vaak hebt u met gemak uw eten kunnen doorslikken?

□ nooit □ zelden □ af en toe □ vaak □ (bijna) altijd

4. Hoe vaak hebt u uw tanden of kunstgebit als een probleem ervaren bij het spreken?

□ nooit □ zelden □ af en toe □ vaak □ (bijna) altijd

5. Hoe vaak hebt u zonder ongemak kunnen eten wat u wilde?

□ nooit □ zelden □ af en toe □ vaak □ (bijna) altijd

6. Hoe vaak hebt u uw contact met anderen beperkt door de conditie van uw tanden of kunstgebit?

□ nooit □ zelden □ af en toe □ vaak □ (bijna) altijd

7. Hoe vaak was u tevreden of blij met hoe uw tanden, tandvlees of kunstgebit eruit zien?

□ nooit □ zelden □ af en toe □ vaak □ (bijna) altijd

8. Hoe vaak hebt u medicijnen gebruikt tegen pijn of ongemak in het gebied van uw mond?

□ nooit □ zelden □ af en toe □ vaak □ (bijna) altijd

9. Hoe vaak hebt u zich zorgen gemaakt om problemen met uw tanden, tandvlees of kunstgebit?

□ nooit □ zelden □ af en toe □ vaak □ (bijna) altijd

10. Hoe vaak voelde u zich nerveus of in verlegenheid gebracht door problemen met uw tanden, tandvlees of kunstgebit?

□ nooit □ zelden □ af en toe □ vaak □ (bijna) altijd

11. Hoe vaak hebt u zich ongemakkelijk gevoeld bij het eten in gezelschap van anderen door problemen met uw tanden of kunstgebit?

□ nooit □ zelden □ af en toe □ vaak □ (bijna) altijd

12. Hoe vaak waren uw tanden of uw tandvlees gevoelig voor hitte, kou of snoep?

□ nooit □ zelden □ af en toe □ vaak □ (bijna) altijd
