# Supplementary material for: Validation of a simplified oral indicator for home care nurses to refer older people to dental care professionals
Source: Acta Odontol Scand. 2024 Dec 16;83:42487. doi: 10.2340/aos.v83.42487 (PMC11707687; doi:10.2340/aos.v83.42487)
Supplement: Validation of a simplified oral indicator for home care nurses to refer older people to dental care professionals [file AOS-83-42487-s2.pdf]

Supplementary material has been published as submitted. It has not been copyedited or typeset by Acta Odontologica Scandinavica.

Appendix B -

Distribution of OHAT-NL sum scores of older participants

Sum score	%	n
0	8	11
1	15	21
2	18	26
3	20	28
4	15	21
5	14	20
6	7	9
7	1	2
8	1	1
9	1	2
	100	141
